# Silencing of a putative immunophilin gene in the cattle tick *Rhipicephalus (Boophilus) microplus *increases the infection rate of *Babesia bovis *in larval progeny

**DOI:** 10.1186/1756-3305-2-57

**Published:** 2009-11-20

**Authors:** Reginaldo G Bastos, Massaro W Ueti, Felix D Guerrero, Donald P Knowles, Glen A Scoles

**Affiliations:** 1 Program in Vector-Borne Diseases, Department of Veterinary Microbiology and Pathology, Washington State University, Pullman, WA 99164-7040, USA; 2 USDA-ARS, Knipling Bushland US Livestock Insect Research laboratory, 2700 Fredericksburg Road, Kerrville, TX 78028, USA; 3 Animal Disease Research Unit, USDA-ARS, Washington State University, 3003 ADBF, P.O. Box 646630, Pullman, WA 99164-6630, USA

## Abstract

**Background:**

The cattle tick *Rhipicephalus (Boophilus) microplus *is involved in the transmission of the protozoan *Babesia bovis*, the etiological agent of bovine babesiosis. Interactions between ticks and protozoa are poorly understood and the investigation of tick genes that affect tick fitness and protozoan infection can set the stage for dissecting the molecular interactions between the two species.

**Results:**

In this study, RNA interference was used to silence *R. microplus *genes that had been previously shown to be up-regulated in response to *B. bovis *infection. The silencing of a putative immunophilin gene (*Imnp*) in female ticks fed on a calf acutely infected with *B. bovis *decreased the hatching rate and survival of larval progeny. Interestingly, *Imnp *was up-regulated significantly in ovaries of *R. microplus *in response to *B. bovis *infection and its silencing in female ticks significantly increased the infection rate of the protozoan in larval progeny. The results also showed that the silencing of a putative Kunitz-type serine protease inhibitor (*Spi*) gene and a putative lipocalin (*Lpc*) gene decreased the fitness of *R. microplus *females, but had no significant effect on the infection rate of *B. bovis *in larval progeny.

**Conclusion:**

The silencing of the *Imnp*, *Spi *or *Lpc *genes decreased the fitness of *R. microplus *females fed on a calf during acute *B. bovis *infection. The *Imnp *gene data suggest that this putative immunophilin gene is involved in the defense system of *R. microplus *against *B. bovis *and may play a role in controlling the protozoan infection in tick ovaries and larval progeny.

## Background

Ticks are obligate hematophagous ectoparasites that can affect human and animal health both directly by blood feeding and indirectly by transmitting pathogens. The cattle tick *Rhipicephalus (Boophilus) microplus *is an economically important ectoparasite of bovines implicated in the transmission of the apicomplexan protozoan *Babesia bovis*, the etiological agent of bovine babesiosis (also known as tick fever) [1]. Adult females *R. microplus *acquire *B. bovis *merozoites by ingesting blood from an infected bovine and pass the protozoan transovarially to their larvae progeny that can transmit *B. bovis *sporozoites to cattle during subsequent feeding [1-3]. The control of bovine babesiosis relies on the control of tick populations and the use of live attenuated vaccines in some endemic areas [1,3]. The control of *R. microplus *is based on the use of acaricides and to a lesser extent by vaccination [4,5]; however the efficacy of commercial anti-tick vaccines is inconsistent in different regions of the world and the recent development of tick populations resistant to acaricides represents a serious threat to the cattle industry [6,7]. Additionally, the reemergence of *R. microplus *in areas that had been considered to be free of this tick, such as the area outside the permanent quarantine zone in south Texas, US, is causing concerns about the reintroduction of *B. bovis *into regions that are currently free of bovine babesiosis [8]. Exposure of naïve cattle to *B. bovis *would lead to significant mortality since no protective immunity is present in the US population. A better understanding of the interactions among ticks, protozoan and cattle is required for the development of epidemiological models and strategies to prevent the reinvasion of *R. microplus *into zones free of this tick and the introduction of *B. bovis *into non-endemic areas.

Interactions between *R. microplus *and *B. bovis *were recently investigated using a proteomic approach and differential protein expression was demonstrated in female ticks in response to *B. bovis *infection [9,10]. Up-regulation of proteins encoded by a putative immunophilin (*Imnp*) gene and a putative Kunitz-type serine protease inhibitor (*Spi*) gene was demonstrated in ovaries of ticks fed on cattle infected with *B. bovis*. Immunophilin proteins, also known as cyclophilin, are linked to multiple cellular processes, such as protein folding, trafficking and defense mechanisms [11], whereas serine protease inhibitors are involved in blocking blood coagulation in *R. microplus *[12]. Preliminary analyses showed that another *R. microplus *gene, which encodes for a putative lipocalin (*Lpc*), was up-regulated in the guts of female ticks fed on *B. bovis*-infected cattle (F.D. Guerrero, unpublished data). Lipocalin proteins, also known as histamine-binding proteins, play a role in the synthesis and transport of small molecules implicated in regulatory functions [13].

Cellular and molecular responses of *R. microplus *to *B. bovis *infection are poorly understood, but it is reasonable to consider that adaptation between the two species may have led the ticks to develop defenses against the protozoan infection. Therefore, it is rationale to assume that *R. microplus *genes up-regulated during *B. bovis *infection may play a role in tick fitness and defense against the protozoan. In the present study we tested the hypothesis that the silencing of the *Imnp*, *Spi *or *Lpc *genes through RNA interference (RNAi) affects the fitness of *R. microplus *females and the rate of infection with *B. bovis *in larval progeny. The data showed that the silencing of the *Imnp*, *Spi *or *Lpc *genes decreased the fitness of female ticks; however only the silencing of *Imnp *had a significant effect on increasing *B. bovis *infection in larval progeny.

## Results

### Transcription level and gene silencing

**Figure 1:**
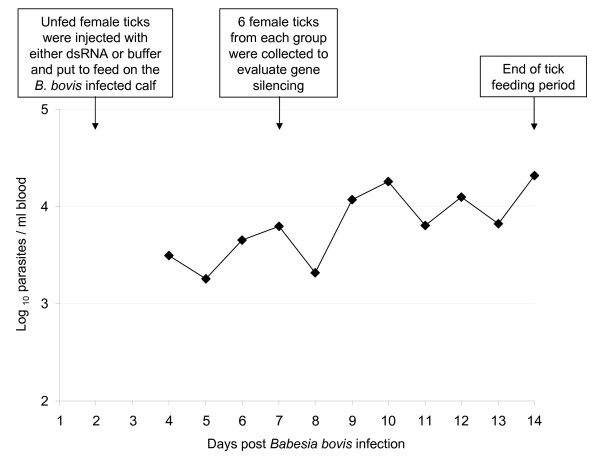
**Parasitemia of *B. bovis *during acute infection and timeline indicating the time-point for double stranded RNA injection, collection of ticks to assess gene silencing, and feeding period**. The *B. bovis *parasitemia was determined by quantitative real-time PCR and presented as log_10 _parasites per ml of peripheral blood.

The level of expression and silencing of the *Imnp*, *Sp*i and *Lpc *genes were investigated by quantitative real-time RT-PCR (qRT-PCR) in tick gut and ovary samples collected at day 5 of feeding (Figure 1). As a first step for standardization of the qRT-PCR, actin, tubulin, glucose 6-phosphate dehydrogenase (*G6PDH*), and phospholipid-hydroperoxide glutathione peroxidase (*PHGPx*) were evaluated as tick reference gene candidates for data normalization. The geNorm [14] analyses showed that none of reference gene candidates were stably expressed and therefore these candidates were considered inadequate for qRT-PCR normalization (data not shown). Consequently, the qRT-PCR was normalized to the total amount of RNA used to generate the cDNA and the transcription level was calculated as a relative expression using the formula: Relative expression_(sample) _= 2^[*Ct*(control)-*Ct*(sample)]^, where the control is the lowest *Ct *value for a given gene of interest (GOI). Efficiency of amplification of the qRT-PCR for the GOI ranged from 102 to 109% and melt curve analyses showed the absence of primer dimers and nonspecific amplification.

**Figure 2:**
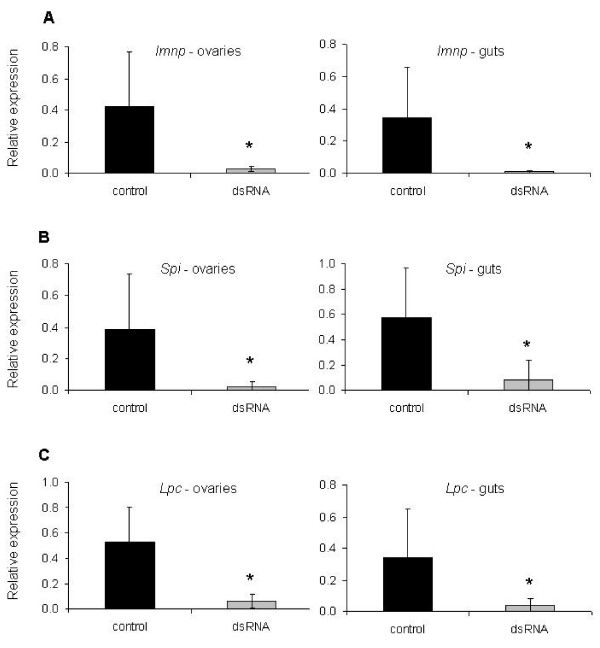
**Transcript level of the imunophilin (*Imnp*) (A), serine protease inhibitor (*Spi*) (B) and lipocalin (*Lpc*) (C) genes in ovaries and guts of six partially engorged *R. microplus *females injected with dsRNA (grey bar) or buffer (black bar) and fed on a calf acutely infected with *B. bovis***. *Imnp *was silenced 92.9% (± 3.1%) and 93.6% (± 2.8%) in ovaries and guts, respectively. *Spi *was silenced 93.4% (± 7.0%) and 85.3% (± 26.4%) in ovaries and guts, respectively. *Lpc *was silenced 81.5% (± 9.6%) and 86.8% (± 12.5%) in ovaries and guts, respectively. The data represent mean values of relative expression of the genes of interest. Asterisk (*) indicates difference between control and dsRNA-injected groups as determined by *t*-test (*P *< 0.05).

To assess gene silencing, female ticks were injected with dsRNA identical to the *Imnp*, *Spi *or *Lpc *genes and fed on a calf acutely infected with *B. bovis*, and at day 5 of feeding, 6 biological replicates of individual ovaries and guts were examined by qRT-PCR (Figure 1 and 2). The *Imnp *gene was silenced 92.9% (± 3.1%) and 93.6% (± 2.8%) in ovaries and guts of dsRNA-injected ticks, respectively, and its relative expression decreased more than 14 times (*P *= 0.0150) in ovaries and more than 32 times (*P *= 0.0406) in guts. The *Spi *gene was silenced 93.4% (± 7.0%) and 85.3% (± 26.4%) in ovaries and guts of dsRNA-injected ticks, respectively, and the relative expression of this gene decreased more than 15 times (*P *= 0.0234) in ovaries and more than 6 times (*P *= 0.0213) in guts. The *Lpc *gene was silenced 81.5% (± 9.6%) and 86.8% (± 12.5%) in ovaries and guts of dsRNA-injected ticks, respectively, and its relative expression decreased more than 8 times in ovaries (*P *= 0.0021) and guts (*P *= 0.0284).

### Biological effect of gene silencing

**Figure 3:**
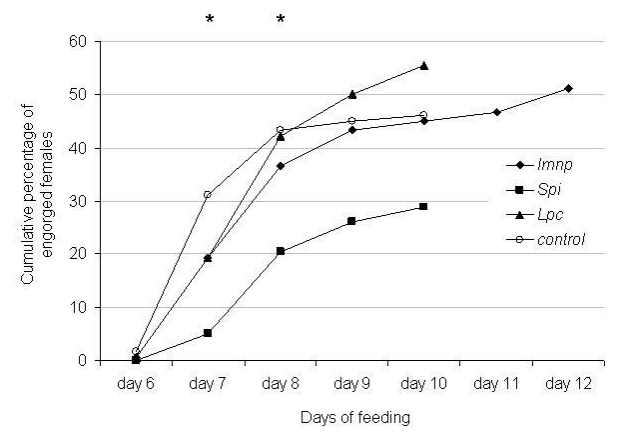
**Cumulative percentage of engorgement of *R. microplus *females injected with dsRNA to silence the immunophilin (*Imnp*) (black lozenge), serine protease inhibitor (*Spi*) (black square) or lipocalin (*Lpc*) (black triangle) genes and control females (white circle)**. The graphic shows the effect of gene silencing on engorgement and survival of female ticks throughout the feeding period. Asterisk (*) indicates difference between the control and the *Spi *dsRNA-injected groups as determined by Chi-squared test (*P *< 0.01).

To investigate the biological effect of gene silencing, dsRNA-injected female ticks were fed on a calf during acute *B. bovis *infection and tick fitness was evaluated (Table 1). Fifty two out of 180 female ticks (28.8%) fed to repletion in the *Spi *silenced group whereas 83 out of 180 females (46%) fed to repletion in the control group demonstrating that the silencing of the *Spi *gene significantly decreased (*P *= 0.0011) the number of engorged females. In contrast, the silencing of the *imnp *and *Lpc *genes had no effect on the number of engorged females. The average weight of engorged females in the *Lpc *silenced group was 364.3 mg and significantly higher (*P *= 0.0167) than the average weight of engorged females in the control group (342.4 mg). However, the silencing of *imnp *and *Spi *did not affect the weight of engorged females. Overall, the rate of oviposition was not affected by gene silencing. The average weight of egg masses in the *Spi *silenced group was 117.8 mg and significantly lower (*P *= 0.0329) than the average of weight of egg masses in the control group (141.1 mg). The silencing of *Imnp *and *Lpc *had no effect on the weight of egg masses. Fifty seven out of 82 egg masses (69.5%) hatched in the *Imnp *silenced group whereas 62 out of 93 egg masses (66.6%) hatched in the *Lpc *silenced group. In comparison, 74 out of 82 egg masses (90.2%) hatched in the control group showing that the silencing of *Imnp *or *Lpc *decreased significantly the percentage of hatching (*P *= 0.0016 and *P *= 0.0002, respectively). The silencing of the *Spi *gene did not affect the percentage of hatching. The silencing of *Imnp *decreased the percentage of larvae survival significantly (*P *< 0.0001) and 11 out of 57 larval progeny (19.3%) died 45 days after hatching whereas 100% (n = 74) of the larval progeny of the control group survived in the same period. In contrast, the silencing of *Spi *or *Lpc *showed no effect on the percentage larval survival. Additionally, the cumulative percentage of engorgement during the feeding period was significantly lower in the *Spi *silenced females than the control females at days 7 and 8 of tick feeding (*P *< 0.0001 and *P *= 0.0004, respectively) (Figure 3). The cumulative percentage of engorgement in the *Imnp *and *Lpc *silenced groups was not significantly different from the control group.

**Table 1:** Biological effect of gene silencing in *R. microplus *females fed on a calf during acute *Babesia bovis *infection.

Experimental groups	Percentage of engorged females	Weight (mg)of engorged females (SD)	Oviposition rate	Egg mass (mg) (SD)	Percentage of hatching	Percentage of larvae survival
Control	46.1% (83/180)	342.4 (± 0.567)	98.7% (82/83)	141.1 (± 0.423)	90.2% (74/82)	100% (74/74)
*Imnp *dsRNA	51.1% (92/180)	349.3 (± 0.907)	89.1% (82/92)	132.3 (± 0.662)	69.5%^1 ^(57/82)	80.7%^1 ^(46/57)
*Spi *dsRNA	28.8%^1^ (52/180)	354.1 (± 0.615)	98.0% (51/52)	117.8^2 ^(± 0.552)	94.1% (48/51)	97.9% (47/48)
*Lpc *dsRNA	55.5% (100/180)	364.3^2 ^(± 0.662)	93.0% (93/100)	128.3 (± 0.558)	66.6%^1 ^(62/93)	96.7% (60/62)

*Imnp *- a putative immunophilin gene; *Spi *- a putative Kunitz-type serine protease inhibitor; *Lpc *- a putative lipocalin gene

^1 ^Chi-squared test (*P *< 0.01)

^2 ^*t *test (*P *< 0.05)

### *B. bovis *infection in larval progeny

**Figure 4:**
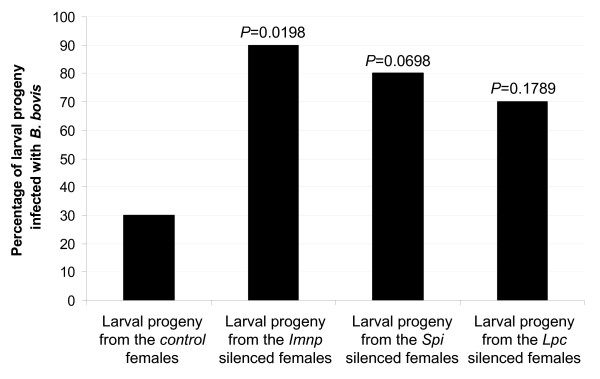
**Infection rate of *B. bovis *in larval progeny from gene silenced *R. microplus *females**. *Imnp *- a putative immunophilin gene; *Spi *- a putative Kunitz-type serine protease inhibitor; *Lpc *- a putative lipocalin gene. The percentages of larval progeny infected with *B. bovis *were compared by Chi-squared test and *P *values for each experimental group are shown.

The larval stage of *R. microplus *is the only tick stage implicated in the natural transmission of *B. bovis *[1]. Thus, in this study the *Imnp*, *Spi *or *Lpc *genes were silenced in *R. microplus *females fed on a *B. bovis*-infected calf and the larval progeny examined for the presence of the protozoan (Figure 4). The infection rate of *B. bovis *was significantly higher (*P *= 0.0198) in larval progeny from the *Imnp *silenced females than in larval progeny from the control group. As a result, 90% of the samples of larval progeny from the *Imnp *silenced females were infected with *B. bovis *whereas 30% of the samples of larval progeny from the control group were infected with the protozoan. Although not significant, there was a tendency of higher infection ratio of *B. bovis *in larval progeny from the *Spi *(*P *= 0.0698) and *Lpc *(*P *= 0.1789) silenced females than in the larval progeny from the control females, and consequently 80% and 70% of larval progeny from the *Spi *and *Lpc *silenced groups, respectively were infected with the protozoan.

### Effect of *B. bovis *infection on the pattern of expression of the *Imnp*, *Spi *and *Lpc *genes

**Figure 5:**
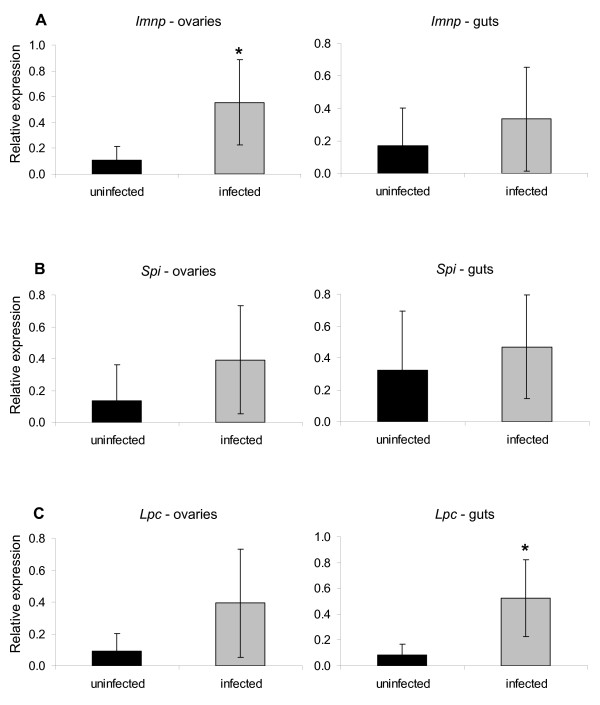
**Transcript level of the imunophilin (*Imnp*) (A), serine protease inhibitor (*Spi*) (B) and lipocalin (*Lpc*) (C) genes in ovaries and guts of six partially engorged *R. microplus *females fed on either an uninfected calf (black bar) or a *B. bovis*-infected calf (grey bar)**. The data represent mean values of relative expression of the genes of interest. Asterisk (*) indicates difference between dsRNA-injected and control groups as determined by *t*-test (*P *< 0.05).

The effect of *B. bovis *infection on the pattern of expression of the *Imnp*, *Spi *and *Lpc *genes was investigated in ovaries and gut of *R. microplus *females (Figure 5). *Imnp *was significantly up-regulated (*P *= 0.0142) 5 times in ovaries of ticks fed on the infected calf compared to ticks fed on the uninfected calf. Although not significant, there was a tendency of up-regulation of *Imnp *in guts of females fed on the *B. bovis*-infected calf. *B. bovis *infection did not significantly affect the level of expression of the *Spi *gene, despite the up-regulation in ovaries and guts of females fed on the infected calf compared to ticks fed on the uninfected calf. The *Lpc *gene was significantly up-regulated (*P *= 0.0049) more than 6 times in guts of female ticks fed on the *B. bovis*-infected calf compared to females fed on the uninfected calf. Even though *Lpc *was up-regulated more than 4 times in ovaries of ticks fed on the infected calf, the difference was not statistically significant.

## Discussion

In the present study we demonstrate that the silencing of *Imnp*, a putative immunophilin gene, decreased the hatching rate and survival of larval progeny of *R. microplus *females fed on a calf acutely infected with *B. bovis*. Importantly, the silencing of *Imnp *significantly increased the infection rate of *B. bovis *in larval progeny. In contrast, the silencing of *Spi *and *Lpc*, a putative serine protease inhibitor gene and a putative lipocalin gene, respectively, decreased the fitness of *R. microplus *females, but had no significant effect on the infection rate of the protozoan in larval progeny. Based on the *Imnp *gene data, we accepted the hypothesis that the silencing of this putative immunophilin gene affects the fitness of *R. microplus *females and the infection rate of *B. bovis *in larval progeny.

It has been demonstrated that the silencing of the immunophilin/cyclophilin gene in *Haemaphysalis longicornis *ticks decreases the weight of engorged females and egg masses [15]. Here we show that the silencing of the *R. microplus Imnp *gene in tick females had no effect on the weight of engorged females and egg masses; however, it decreased hatching and larvae survival suggesting that this gene plays a role in tick fitness. Additionally, *Imnp *was significantly up-regulated in ovaries of ticks fed on the *B. bovis*-infected calf compared to ticks fed on an uninfected calf. This data supports the previous observation of up-regulation of the protein encoded by the *Imnp *gene in ovaries of *R. microplus *in response to *B. bovis *infection [9]. Immunophilins are cyclosporine-binding proteins (also known as cyclophilins) that have been described in mammals, plants, insects, fungi and bacteria [11]. Immunophilins contain a peptiyl-prolyl isomerase (PPIase) domain that interconvert the *cis *and *trans *isomers of peptide bonds of the amino acid proline and are involved in multiple cellular processes, such as protein folding, trafficking and defense mechanisms [11,16]. Numerous putative immunophilin/cyclophilin genes have been identified in the *Ixodes scapularis *tick genome http://iscapularis.vectorbase.org/; however, no functional study has been reported yet. The deduced amino acid sequence of the *Imnp *gene used in the present study contains a PPIase domain (amino acid 49 to 208, Pfam prediction, E value = 1.80e-117), and has 90% and 82% identity with the immunophilyn/cyclophilin proteins of *H. longicornis *and *I. scapularis*, respectively (data not shown), suggesting that *Imp *encodes for a putative *R. microplus *immunophilin gene.

The silencing of the *Spi *gene reduced the number of engorged females causing a delay in the engorgement time suggesting that this gene is involved in the processes of feeding and digestion in *R. microplus *as previously described for other members of serine protease inhibitors [12,17]. Although not statistically significant, the up-regulation of *Spi *in ovaries of ticks fed on a calf acutely infected with *B. bovis *may have biological relevance and explain the up-regulation of the Spi protein in ovaries of *R. microplus *fed on cattle infected with the protozoan described elsewhere [9]. It has been shown that a Kunitz-type serine protease inhibitor is involved in the defense mechanisms of adult *Dermacentor variabilis *ticks against *Rickettsia montanensis *[18]. Here we showed that silencing of the *Spi *gene in female ticks did not result in a significant increase of *B. bovis *infection in *R. microplus *larvae. Despite the fact that the serine protease inhibitor is a large protein family that includes several members, these data suggest that different tick species or tick stages may use different strategies to control *Babesial *or *Rickettsial *infection. Serine proteases are part of the clotting mechanisms of mammals and serine protease inhibitors play a role in blocking blood coagulation. Considering hematophagous ectoparasites, the blood coagulation cascade of the mammalian host is essentially inhibited by protease inhibitors [19]. Several serine protease inhibitors have been identified in *R. microplus *and shown to inhibit major procoagulant enzymes, such as thrombin, trypsin and plasmin [12,17]. The N-terminal amino-acid sequence of the protein encoded by the *R. microplus Spi *gene has 85% identity with the *R. microplus *Kunitz-type serine protease inhibitor (GeneBank accession no. P83609) and conservation among cysteine position, suggesting that *Spi *encodes for a putative Kunitz-type serine protease inhibitor [9]. The *Imnp *and *Spi *genes were up-regulated in *R. microplus *ovaries and guts, respectively in response to *B. bovis *infection and their silencing affected the fitness of the ticks and rate of *B. bovis *infection in larval progeny. Despite the fact that there is no direct association between the putative function of the two genes, it is reasonable to speculate that concurrent silencing of these genes may have a synergetic effect in controlling the protozoan infection in ticks and further experiments are needed to investigate this hypothesis.

The *R. microplus Lpc *gene encodes for a putative lipocalin protein which contains a histamine-binding domain from residue 35 to 180 (Pfam E-value: 1.30e-29) (data not shown). The lipocalin protein family is a large group of extracellular proteins which exhibit a variety of functions that include transport of small molecules, prostaglandin synthesis, smell reception and regulation of immune response [13,20]. It has been shown that the earlier detachment of *R. microplus *larvae from resistant cattle is related to the release of histamine at the attachment site [21]. The silencing of tick genes that encode for histamine-binding proteins has shown to have a marked effect on tick fitness [22,23]. Here we show that the silencing of the *R. microplus Lpc *gene had no affect on the duration of the tick feeding period, but it decreased the weight of engorged females and the percentage of hatching demonstrating that this putative lipocalin gene affects tick fitness. *Lpc *was significantly up-regulated in gut of ticks fed on a calf during acute *B. bovis *infection compared to ticks fed on an uninfected calf, confirming previous observation (F.D. Guerrero, unpublished data). High-affinity histamine-binding proteins involved in the suppression of host inflammatory response during tick feeding have been identified in saliva of *Rhipicephalus appendiculatus *ticks [24]. It has been shown that the bovine immune system produces inflammatory mediators, such as nitric oxide and cytokines, in response to acute *B. bovis *infection [25]. Considering that lipocalin proteins suppress inflammation, it is tempting to speculate that the *Lpc *gene may play a role in *R. microplus *gut by suppressing the inflammatory mediators present in the blood meal from acutely *B. bovis*-infected cattle.

*R. microplus *females acquire *B. bovis *infection and pass the protozoan transovarially to their larval progeny which will transmit it to naïve cattle [1]. Therefore, it was of interest to silence genes that had been previously demonstrated to be up-regulated in female ticks in response to *B. bovis *infection and investigate the presence of the protozoan in larval progeny. It has been shown in *Anopheles gambiae *mosquito that targeting up-regulated genes, such as C-type lectin and leucine-rich genes, for silencing affected the infection rate of *Plasmodium berghei *in mosquitoes [26]. We show that the infection rate of *B. bovis *in larval progeny from the *Imnp *silenced females was significantly higher than the control group suggesting that this gene plays a role in defending *R. microplus *against the protozoan infection. Thirty percent of the larvae samples of the control group were infected with *B. bovis*, showing that only a minor proportion of larval progeny is transovarially infected when *R. microplus *females fed on an acutely infected calf as described elsewhere [27]. In contrast, 90% of the larval progeny samples from the *Imnp *silenced group were infected with *B. bovis *demonstrating the biological relevance of this gene as an antagonist of the protozoan infection. It has been shown that infection with *Babesia spp *can affect tick fitness and the severity of these effects is related to the degree of parasitemia [1]. In this study, clinical signs and parasitemia may have affected the tick fitness; however the overall biological impact cannot be solely attributed to *B. bovis *infection, considering the differences in tick fitness observed between the silenced and control groups.

RNAi has been widely used to knock-down gene expression in a sequence-specific manner, making it a powerful tool for investigating gene function. However, off-target effects have been described in numerous species [28-30] and cannot be entirely ruled out as the cause of the results observed in this experiment. The *R. microplus *genome sequence is not available [31] and the alignment analyses are restricted to sequences listed in databases. Blast analysis of the dsRNA sequences used in this study did not reveal significant homology to any known tick sequence other than the GOI. Additionally, the GOI were specifically silenced by the dsRNA injection. Taken together, these two aspects support the data and represent the best possible strategy to make solid scientific observations regarding biological effect of gene silencing in *R. microplus*.

In summary, we show that the silencing of *Imnp*, *Spi *or *Lpc*, three novel genes of *R. microplus*, decreased the fitness of females ticks fed on a calf during acute *B. bovis *infection. Notably, the *Imnp *gene was significantly up-regulated in ovaries of *R. microplus *in response to infection and its silencing in female ticks increased significantly the infection rate of *B. bovis *in larval progeny. Collectively, these data demonstrate that *Imnp *acts as an antagonist against *B. bovis *infection suggesting that this gene may play a role in controlling the transovarial transmission of the protozoan in *R. microplus*. These observations may also account for the presence of a tick defense system to control protozoan infection and further studies are needed to characterize the mechanism used by the *Imnp *gene to affect *B. bovis *infection in *R. microplus*.

## Materials and methods

### Cattle, ticks and protozoan

Two Holstein calves 3-4 months of age that tested negative for *B. bovis *by nested PCR [27] were used in this study. The animals were maintained according to protocols approved by the University of Idaho Institutional Animal Care and Use Committee. One calf remained uninfected throughout the experiment and the other calf was inoculated intravenously with approximately 1.4 × 10^8 ^*B. bovis*-infected erythrocytes (T2Bo strain) [32]. Parasitemia in peripheral blood was examined by quantitative real time PCR to amplify the single copy *B. bovis *msa-1 gene. For the msa-1 quantitative real time PCR, specific primers (5' gatgcgtttgcacatgctaag 3' and 5' cgggtacttcggtgctctca 3') and probe (FAM 5'-cacgctcaagtaggaaattttgttaaacctgga-3' TAMRA) were used following the assay conditions described elsewhere [27]. At day 6 after the *B. bovis *inoculation, the infected calf started showing clinical signs of acute babesioses marked by a decrease of 20% in packed cell volume and temperature above 39°C. The La Minita strain of *R. microplus *used in this study has been maintained at the University of Idaho Holm Research Center since 1999 as previously described [33]. To obtain uninfected and unfed adult ticks, approximately 40,000 larvae (from 2 g of eggs) were placed under a cloth patch on the uninfected calf. After 13-14 days, engorged nymphs were manually removed with forceps and incubated in vitro at 26°C to induce molting to adult stage. Unfed adult ticks were sexed and the females used for gene expression and silencing experiments. The biological effect of gene silencing was investigated in female ticks fed until repletion on the *B. bovis*-infected calf whereas the pattern of gene expression was examined in females fed until repletion on either the *B. bovis*-infected calf or the uninfected calf.

### Target genes and synthesis of double stranded RNA

Specific primers were designed based on the cDNA sequences of the *Imnp *(TC12157), *Spi *(TC9311) and *Lpc *(TC8123) genes described in the *R. microplus *Gene Index Project http://compbio.dfci.harvard.edu/tgi/tgipage.html (Table 2). PCR products from each one of the GOI were individually cloned into pCR^® ^4-TOPO^® ^(Invitrogen, Carlsbad, CA, USA), sequenced and analyzed *in silico *for the presence of putative small interfering RNA (siRNA) by the algorithm siRNA Target Finder (Ambion, Austin, TX, USA). A fragment with approximately 400 bp from each GOI containing the highest number of putative siRNA was amplified by PCR, cloned into pCR^®^II-TOPO^® ^(Invitrogen) and used as template for the dsRNA synthesis (Table 2). The MEGAscript^® ^Transcription Kit (Ambion) was used for the dsRNA synthesis following the manufacturer's protocol. The dsRNA molecules were checked by electrophoresis on agarose gel, quantified by spectrophotometry and kept at -20°C until used for tick injection.

**Table 2:** Gene identification, primers, gene region amplified, amplicon size and PCR annealing temperature.

Gene identification (purpose)	TC number* or GenBank identification	Forward primer (5'-3')	Reverse primer (5'-3')	Size (bp)	Annealing temperature (°C)
*Imnp *(full-length)	TC12157	gcagaattaactatcgcaaa	tttgtgtggtggaaatgaa	928	58
*Imnp *(dsRNA synthesis)	TC12157	agacttcacgaatcacaa	taagaagaaaggaaaagttg	380	58
*Imnp *(real-time PCR)	TC12157	ggttacttgtgtggttgt	gtgtggtggaaatgaat	64	60
*Spi *(full-length)	TC9311	caattcatcctgtgtattctcg	aacccattcgcctgttacg	1652	58
*Spi *(dsRNA synthesis)	TC9311	caattcatcctgtgtattctcg	caacaacttcgtgactct	396	58
*Spi *(real-time PCR)	TC9311	taatagcgagggaagtag	caccaaggagaccatttc	135	60
*Lpc *(full-length)	TC8123	accccgaatccctgtgtt	cagtgccgaccaaagtaagg	731	58
*Lpc *(dsRNA synthesis)	TC8123	gagaagataggaaatgtgta	tctctgttgtagacgaagt	379	58
*Lpc *(real-time PCR)	TC8123	aaggagggaagaatacac	ctcaagtttcaaggacag	134	60
Actin (real-time PCR)	AY255624	tcctctccttccttctac	aaagttctgttcgtcgt	103	60
tubulin (real-time PCR)	AA257910	tgtgccccgtgccgtattt	ggcaccagagcgaacct	64	60
*G6PDH *(real-time PCR)	EU595881	ttctctctctcctcagtg	gaagatgtgttgttgtcc	88	60
*PHGPx *(real-time PCR)	DQ180067	accaacaagaactacacg	cttctttatgtctgcctca	131	60

*Imnp *- a putative immunophilin gene; *Spi *- a putative Kunitz-type serine protease inhibitor; *Lpc *- a putative lipocalin gene; TC - tentative consensus; * the *R. microplus *Gene Index Project http://compbio.dfci.harvard.edu/tgi/tgipage.html.

### Injection of ticks with double stranded RNA

Freshly molted unfed females were used for the dsRNA injection. Three groups with 200 female ticks each received a single injection with dsRNA identical to the target region of the *Imnp*, *Spi *or *Lpc *genes. Another group also with 200 female ticks was injected with buffer (0.1 mM EDTA) and used as control. For injection, individual females were placed on double-sided tape with the ventral side upwards and injected with either 1 μl of dsRNA (approximately 1 × 10^11 ^molecules dissolved in buffer 0.1 mM EDTA) or 1 μl of EDTA buffer through the coxal membrane at the base of the 4th leg on the right ventral side. Injections were accomplished using a 10 μl syringe with a 33 gauge needle (Hamilton, Bonaduz, Switzerland) and the microprocessor controlled UMP3 injection pump apparatus (World Precision Instruments, Berlin, Germany). After the injection, the female ticks of each group plus an equal number of males were placed under individual stockinet sleeves glued to the side of the *B. bovis*-infected calf (2 days after the experimental *B. bovis *infection). At day 5 post-dsRNA injection, 6 partially engorged females from each group were dissected and individual ovaries and guts collected in RNAlater (Ambion) to evaluate gene silencing.

### Evaluation of tick fitness

After the dsRNA injection, individual stockinet sleeves containing each experimental group were monitored daily for the presence of engorged females. The effect of gene silencing on engorgement and survival of female ticks was investigated by assessing the cumulative percentage of engorged females during the feeding period. Individual engorged females were weighed and put in 24-well plates at 26°C to assess oviposition. At day 14 after the beginning of oviposition, eggs laid by individual females were weighed, put in individual vials and incubated at 26°C to evaluate hatching. Hatching was evaluated at 30 days after the egg masses were collected and was defined as the presence of any larvae in eggs from each individual female. The larval progeny was maintained in individual vials at 26°C for 45 days and the larvae survival was determined as the presence of any live larvae in larval progeny from individual females.

### Gene expression and silencing

A qRT-PCR was standardized for each GOI to assess gene expression and silencing. Total RNA was extracted from individual ovary or gut samples using the RNAqueous^® ^Kit (Ambion) according to the manufacturer's protocol and quantified by Qubit^® ^Fluorometer (Invitrogen). The RNA samples were treated with DNase I (Invitrogen) following the manufacturer's protocol and 200 ng of total RNA of each sample were used for cDNA synthesis using the Superscript^® ^Vilo™ cDNA Synthesis Kit (Invitrogen) following the manufacturer's protocol. For the qRT-PCR, specific primers for each GOI were designed to avoid the gene region used to synthesize the dsRNA (Table 2). For the standardization of the qRT-PCR, the *R. microplus *actin, tubulin, *G6PDH *and *PHGPx *genes were evaluated as tick reference gene candidates (Table 2). The geNorm applet [14] was used to examine the stability of expression of the reference gene candidates. The qRT-PCR for the GOI and reference gene candidates were performed in a CFX96™ Real-Time PCR Detection System using the Express SYBR^® ^GreenER™ Supermix Kit (Invitrogen). The cycling conditions consisted of a Uracil-DNA Glycosylase inactivation step of 50°C for 30 sec, initial denaturation of 95°C for 2 min followed by 40 cycles of 95°C denaturation for 15 sec and annealing/extension of 60°C for 45 sec. Reactions were performed in duplicate in 20 μl using 200 nM of each primer and 2 μl of a 1/20 dilution of cDNA as template. An inter-run calibrator was included to assess inter-run variations. The CFX Manager™ Software (Bio-Rad, Hercules, CA, USA) was used to analyze the qRT-PCR data. Efficiency of amplification and melt curve analyses were performed to evaluate analytical sensitivity and specificity of the qRT-PCR for each GOI.

### Infection rate of *B. bovis *in larvae of *R. microplus*

The presence of *B. bovis *in larval progeny of the silenced and control *R. microplus *females was examined by nested PCR with primers to the *B. bovis msa1 *gene as previously described [27]. Briefly, a total of 10 larval progeny samples (containing approximately 100 larvae per sample) of individual females from each experimental group were tested. Genomic DNA (gDNA) was extracted from each larval sample using the Puregene^® ^Genomic DNA Purification Kit (Gentra, Minneapolis, MN, USA) following the manufacturer's protocol and quantified by spectrophotometry. The nested PCR were performed using the HotStarTaq^® ^Plus Master Mix Kit (QIAGEN, Valencia, CA, USA) in 20 μl containing 0.2 μM of each primer. For the first run of the nested PCR, 100 ng of larvae gDNA were used as template with 55°C of annealing temperature. For the second run, 2 μl of a 1/100 dilution of the first PCR run was used as template with 60°C of annealing temperature. The presence of amplifiable DNA was examined by PCR in all negative samples using primers specific for the *R. microplus *actin gene described in Table 2.

### Statistical analyses

The Instat^® ^software (GraphPad Software, Inc., USA), version 3.06, was used to perform the statistical analyses. The relative gene expression and weights of engorged females and egg masses were compared by *t*-test. The percentages of engorged females, oviposition, hatching, larvae survival and infection rate of *B. bovis *were compared by Chi-squared test.

## Competing interests

The authors declare that they have no competing interests.

## Authors' contributions

RGB designed and performed the experiment, and wrote the first draft of the manuscript. MWU designed and performed the experimental procedures involving ticks. FDG suggested the tick genes used in this study and contributed to the experimental design. DPK designed, supervised and acquired funding for the experiment. GAS designed and supervised the experiment and made intellectual contribution regarding data interpretation. All authors reviewed and approved the final version of the manuscript.
